# How to Civilize Elites: Controlling “Foreign Scientists” at a Field Station in the Galápagos Islands

**DOI:** 10.1007/s10739-024-09801-8

**Published:** 2025-01-10

**Authors:** M. Susan Lindee

**Affiliations:** Department of the History and Sociology of Science, University of Pennsylvania, Philadelphia, PA 19104 USA

**Keywords:** Galápagos Islands, Charles Darwin Research Station, Conservation, Collecting

## Abstract

This paper explores the control of visiting “foreign scientists” at the Charles Darwin Research Station (CDRS) after it was established in the Galápagos Islands in 1959. Scholarly accounts of the creation of the Galápagos National Park and of the field station have emphasized their place in an international “land grab,” as leading scientists and conservationists sought to control nature in places around the world that seemed less “civilized” to European thinkers. The actual administrative labor in the early years at this scientific field station, however, in practice struggled to control people widely taken to represent “civilization” in its highest form—European and American scientists. At the research station, European and American (but not Ecuadorian) scientists were the focus of a delicate choreography of discipline and acquiescence, as scientists were courted and refused, welcomed and limited, chastised and supported. Meanwhile CDRS fund-raising appeals promised that the station would control island residents, fishing crews, and invasive species. Such appeals did not mention controlling elite field scientists. Existing historiography has stressed how Western scientists were privileged actors in non-Western nature reserves and parks, their privileges coming at the expense of local communities. But scientists too faced new (quietly implemented) constraints as post-war conservation programs developed, and achieving their compliance with these new rules involved a process I call here “civilizing” elites.

The Charles Darwin Research Station (CDRS), established in 1964 in the Galápagos Islands, resulted from the efforts of a network of jet-setting experts who were negotiating the post-1945 management of nature, particularly in Africa (Congo, Rwanda, Tanzania) but also in other areas. It was imagined and planned over about five years of international discussion, publicized widely in mainstream newspapers and magazines, built in 1962–63, and dedicated in early 1964. In July 1959, as station plans were developing, and with the encouragement of scientists based in Europe and the United States, the government of Ecuador created the Galápagos National Park, which included most of the archipelago’s land surface.

Scientists and conservationists promoting the new station and park hoped to control natural spaces they feared would be degraded or destroyed without “First World” scientific and political management. They drew on an imagined history (a false story about Darwin’s supposed discovery of natural selection on these islands) to seize the Galápagos Islands for “all mankind,” extending colonial practices of controlling nature to places around the world that seemed less “civilized” to European thinkers.

In this paper, I consider the actual labor in the early years at the station, which in practice, struggled to control people widely taken to represent “civilization” in its highest form—European and American scientists. At the CDRS, European and American (but not Ecuadorian) scientists were the focus of a delicate choreography of discipline and acquiescence, as visiting scientists were courted and refused, welcomed and limited, chastised and supported, while the station managed access to a biological field site built around a soaring, emotive, and false historical narrative of Darwin’s experiences there.^1^

Those supporting the creation of the Galápagos research station sought public donations and international support by emphasizing the risks of invasive species and the behavior of local residents, both very real threats to the islands’ ecology. But the field station had no formal authority or role in managing local residents or eliminating invasive species. Managing the local residents was the job of the government of Ecuador, and eliminating invasive species the duty of Ecuadorian-employed wardens of the Galápagos National Park. Scientists approved to work at the station did often address key questions of conservation, invasive species, changes in plant ecology, and other important issues. And the station ran and continues to run a successful breeding and repatriation program for tortoises and iguanas, supports significant public outreach to local residents and visitors, and collaborates with Ecuadorian and international institutions engaged with conservation work in the islands.^2^ It has certainly played a key role in helping to manage threats to the Galápagos Islands ecosystems. But its *unique* role—its quiet, behind the scenes, and absolutely crucial role—has been to control visiting foreign scientists, something no other institution was expected or able to undertake.^3^

My work suggests how delicate this control was and had to be—the station existed seemingly for the benefit of a highly privileged and entitled group, but in fact demanded accountability. The silences around this work were strategic and reasonable—I do not doubt their necessity—but I do suggest that they intersect with the silencing of local stakeholders in the systems of conservation that developed in the mid-20th century. Existing historiography has tended to stress how Western scientists were privileged actors in the rise of conservation around the world, and they certainly were. This paper, however, explores how the same systems that presented the control of less privileged local residents as crucial to conservation (invoking ignorant locals was a common theme in public-facing conservation appeals for support) also, much more quietly, constrained and limited extremely well-informed elites.

Before the CDRS, visiting scientists could take whatever they wanted from the islands, whenever they wanted, particularly in remote and isolated locations where activities could not be observed. After 1964, however, the new station required approval for access and collecting. The CDRS needed to diplomatically persuade visiting experts that their behavior could have dire consequences for the islands, and for the station itself. The CDRS also needed to avoid calling attention to any transgressions by visiting scientists, who were already resented in some circles in Ecuador.

It might have been impolitic to complain publicly about the depredations of scientists, and easier to complain about the damage done by locals and fishing crews, but I propose that blaming local residents might have costs, too.

## Visiting the Galápagos Before CDRS

The original raiders of Galápagos wildlife were 18th-century sailors who collected tortoises for on-board meals; naturalists began to arrive in the 19th century.^4^ After 1800, migrants from Europe and Latin America established four small communities. The newly independent Republic of Ecuador claimed the islands in 1832 and sent some prisoners there, but attempts to productively monetize them—underway during Darwin’s 1835 visit—generally failed. Darwin spent five weeks on the islands in September and October of 1835. He rode a tortoise and consumed roasted tortoise meat while camping with hunters at an upland spring. He caught and dissected an iguana and came so close to a Galápagos hawk that he could push it off a branch with his gun barrel. The finches he collected there he threw together in the same bag, and he did not notice that the mockingbirds differed by island (Darwin 1839; Browne 1995, pp. 300–301).

Other scientific visitors, some inspired by Darwin, came to the islands after the publication of the *Origin* in 1859. In 1868 an expedition out of New York collected materials related to Darwin’s work, and in 1873 Harvard’s anti-evolution skeptic Louis Agassiz visited intending to collect materials that would show Darwin was wrong. Many scientists brought home birds. Darwin left the Galápagos with only thirty-one finches and sixty-four birds total, but later visitors took many more: Habel in 1868 (460 bird specimens), George Baur in 1891 (1100 bird specimens), Charles Harris in 1897 (3075 bird specimens), and the California Academy of Sciences in 1905–1906 (8691 birds alone) (Sulloway 1982a, p. 40; see also Barrow 2000, 2009; James 2017). After 1859, there was an average of one expedition to the Galápagos from various European centers every four years (Sulloway 1982a).

Accounts of early naturalists collecting in the field generally do not describe local constraints on specimen collection. For example in James DelBourgo’s study of collecting by Hans Sloane, Mark Catesby, and others, field naturalists and collectors struggled with the unreasonable demands of sponsors back home, rather than constraints in the field (DelBourgo 2017, pp. 202–257). Local officials do not seem to have restricted biological collection even in places where they controlled access, as Ecuador did in the Galápagos after 1832. Matthew James’s account of the US Galápagos expedition to Ecuador in 1905–1906 describes a group of only eight US scientists extracting 78,000 specimens (almost 10,000 specimens each) during their stay, apparently with no oversight and no locally imposed restrictions. The group thought of themselves as collectors first, and in their letters and records emphasized their plans to maximize the number of exotic species they could capture and kill. James notes that the “unrestrained approach to field collecting” of Rollo Beck and his comrades, “seems foreign or even repulsive to us today,” but they had “a green light to collect as much as they possibly could and carry it back to San Francisco” (James 2017, p. 250).

Field naturalists within the United States did face local constraints relating to game laws and had to apply for licenses to exceed game restrictions. Robert Kohler notes that the explorer-collectors he tracks were “opportunists” who were regularly mistaken for some other sort of person, “often disreputable,” seen perhaps as bank robbers, detectives, deadbeats, or crazy people. “In fact, the chief practices of survey collecting—series shooting and seining—were strictly illegal when performed by sport hunters and fishers. The differences were in intention and purpose” (Kohler 2006, pp. 221–222). This system created some non-intuitive consequences. Hunters sometimes tried to pass as field naturalists so that they could kill more animals, so fish and game wardens who understood the value of scientific collecting nonetheless seem to have become, Kohler notes, “stingier with permits for scientific collecting in the 1910s, not because they thought scientists were abusing the privilege, but because they did not relish explaining to sportsmen why scientists could do what they could not” (Kohler 2006, p. 223).

That scientists tended to be more interested in collecting specimens than in preserving species in the wild is a clear theme throughout Mark Barrow’s (2009) study of the history of extinction, according to which “[m]ost naturalists seemed relatively indifferent to the plight of endangered species and more interested in safeguarding their prerogative to collect rare species than in rescuing them” (Barrow 2009, p. 133). Well into the 1930s, ornithologists and primatologists argued about the proper limits on collecting and debated when exactly collecting threatened species survival. Harold Coolidge, who played key roles in the Galápagos, was criticized for his “overzealous collecting” on a trip to Thailand, Borneo, and Sumatra, where his group killed almost 200 gibbons as part of a project to understand their evolutionary relationship to humans (Barrow 2009, pp. 156–157).

Leaders in the emerging conservation movement wondered if scientists were part of the problem, and in the United States, after 1900, new federal legislation gradually limited (on paper) the activities of field biologists.^5^ Later environmental laws that privileged scientists (making them policy experts) also brought their methods under new scrutiny. By requiring researchers to obtain permits to, as Etienne Benson puts it, “harass, injure, capture, or kill them, or attempt to” (for marine mammals), new legal constraints interpreted scientific field research as potential threats to wildlife (Benson 2012, p. 37). By the 1980s, Benson notes, field biologists faced a “sometimes bewildering thicket of overlapping regulations” and the paperwork alone was daunting (Benson 2012, p. 49). In fact, “[s]cientists were among the architects of the environmental regulatory regime that emerged in the 1960s and 1970s, but by the mid-1970s some of them also saw themselves as its victims” (Benson 2012, p. 49, p. 35; and see discussion Benson 2010, pp. 151–160).

In this context of shifting legislative and cultural responses to the activities and rights of field biologists, the CDRS was one critical site where questions about how to control field scientists were being actively and sometimes uncomfortably negotiated. In the Galápagos, funding sources did not dictate policies in the field, hunting rules were not applicable in a location legally inaccessible to hunters, and endangered species were everywhere.

## Creating the Field Station

Early proposals to preserve the islands as scientific spaces emerged in Ecuador in the 1930s, as the centennial of Darwin’s 1835 visit loomed. Leading Ecuadorian naturalists proposed protecting the islands and building a scientific research station there. Biologists at the University of Guayaquil and in Quito played key roles in this Ecuadorian initiative. In California, the Consul of Ecuador, C. M. Egas, recruited the support of influential scientists including Harry S. Swarth of the California Academy of Sciences (Moore 1935). Perhaps unsurprisingly, Swarth’s proposal for a park and research station submitted to the government of Ecuador in 1933 called for the complete removal of the Galapaguenos, the approximately 1000 people then living on the islands. The islands should become a “wildlife sanctuary and outdoor biological laboratory in honor of Charles Darwin” with no farms, villages, or full-time inhabitants other than scientists.^6^ This removal did not happen, but Ecuador passed laws in 1934 and 1936 to protect some areas as nature reserves. Meanwhile Swarth attracted help from Julian Huxley on a plan to raise funds to endow a scientific research station. Such plans were set aside in the late 1930s, as a desperate world war unfolded (Corley-Smith 1990; Barrow 2009, pp. 176–183).

The United States began to mobilize, and in light of growing concerns about Pacific security, Ecuador agreed to allow the placement of a new US Naval Air Station on the Galápagos Island of Baltra. President Franklin Delano Roosevelt had been to the Galápagos on a cruise in 1938 with Smithsonian Institution biologist Waldo Schmitt, and Roosevelt suggested Schmidt should establish a scientific research station at the air base itself (Corley-Smith 1990; Kramer 2020). 7 This research station was never built, and the airfield was not heavily used during the war, but the Baltra airfield ultimately facilitated scientific work and then tourism in the islands.

After 1945, as the war crisis ended, a network of conservation interests coalesced again around risks to natural settings particularly in less wealthy places around the world. The International Union for the Conservation of Nature (IUCN), created in 1948, grew out of Huxley’s 1946–1948 leadership as Director General of the United Nations Educational, Scientific and Cultural Organization (UNESCO). It was intended to establish international cooperation and provide scientific knowledge and tools to guide conservation. As funding became a challenge, Huxley and his peers created a fund-raising entity, the World Wildlife Fund, to support IUCN and conservation groups in general.^8^

The potential of the Galápagos Islands as a conservation site attracted an impressive list of leading scientists, political appointees, and conservationists. British promoters included evolutionary biologist Huxley and diplomat G. T. Corley-Smith. They were joined by US scientists including ornithologist Robert Bowman and biologist/primatologist Harold Coolidge. The prominent Belgian conservationist Victor Van Straelen was a key contributor, as were the Belgian civil servant/amateur ornithologist Jean Paul Harroy and the French ornithologist Jean Dorst. The major and most visible players in this group were Huxley, Coolidge, and Van Straelen, all of whom were involved in conservation programs around the world.^9^ Huxley was of course scientific royalty, grandson of Darwin’s defender Thomas Henry, and brother of writer Aldous. A key figure in the evolutionary synthesis, Huxley was also the promoter of a form of scientific humanism that he believed could free humanity from the shackles of organized religion (Greene 1990; Waters and Van Helden 1992; Wöbse 2011; Smocovitis 2016; Bashford 2022).

Coolidge was a prominent Harvard primatologist at the Museum of Comparative Zoology who played a key role in the establishment of the World Wildlife Fund and the IUCN (he was president of IUCN 1966–1972), and who conducted major field expeditions collecting primates in Africa, South Asia, and other sites (Barrow 2009, pp. 155–180; Hennessy 2019, pp. 117–130). Van Straelen was a long-time Director of the Musee Royal d’Histoire Naturelle of Belgium, a Professor at the University of Brussels and later Ghent, a member of the Executive Board of IUCN from its inception, and a founding member of the International Commission on National Parks. He played a role in the creation of the first “gorilla sanctuary” in 1926 and in the creation of national parks in Congo and Rwanda (Wöbse 2011; de Bont 2015). They were joined by figures more narrowly engaged with the Galápagos—Corley-Smith was a Cambridge-educated diplomat and birdwatcher who was British Ambassador to Ecuador 1960–1967 (Corley-Smith 2023); Bowman a professor of biology at San Francisco State University whose first trip to the Galápagos came when he was a graduate student and who worked on the finches in the islands (Perlman 2006); and Jean-Paul Harroy (Kiesel and Collette 1995) and Jean Dorst (Vuilleumier 2004) were Galápagos-centered specialists who were important in the development of the Charles Darwin Foundation and the Charles Darwin Research Station.

When the young Austrian ethologist Irenäus Eibl-Eibesfeldt returned from a Galápagos expedition in 1954 disturbed by what he had seen there,^10^ his appeal to the IUCN catalyzed this group into action. Eibl-Eibesfeldt reported that he was shocked by the “persecution of endemic fauna.”^11^ Marine iguanas, sea lions, pigeons, hawks, and many other endemic animals seemed to have become rare, he said, and the skins of fur seals and sea lions, tortoise shells and young tortoises, and even penguins were offered to visitors for sale for “small sums” (Eibl-Eibesfeldt 1958). On Las Plazas Islands he found six sea lions with battered skulls rotting in the sun. His appeal to the IUCN proposed that these discoveries meant that a new Biological Research Station should be set up in the islands, a causal claim which seemed to imply that a research station would be able to stop the destruction of endemic fauna (presumably undertaken by local residents or visiting hunters and fishing crews). In later writings he repeated the idea that a research station could halt damage to the islands (for example in the *UNESCO Courier* in January 1958).

**Fig. 1:**
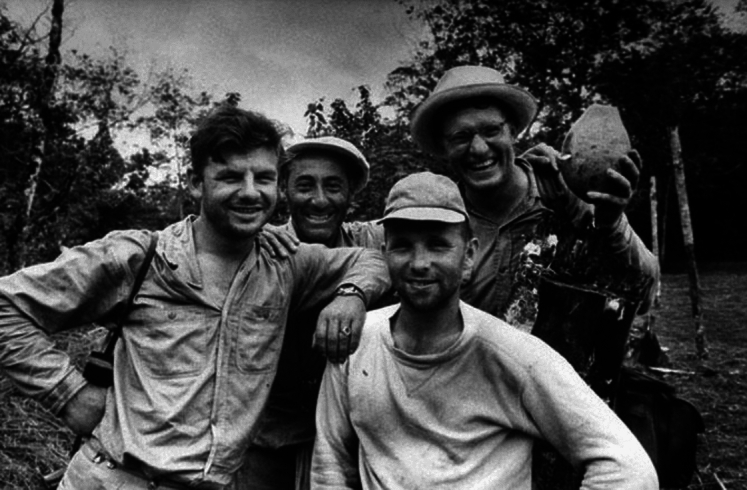
Eible-Eibesfeldt, Bowman, Eisenstadt and Freund in the Galapagos, June 1957. *Life Magazine*

IUCN officials followed up with Ecuadorian government officials, who approved an expedition to identify a site. Their enthusiasm, Elizabeth Hennessy suggests, reflected Ecuadorian concerns about fishing rights in the islands (Hennessy 2019, p. 122). In June 1957 Eibl-Eibesfeldt and Bowman undertook an IUCN mission in the company of *Life* photographer Alfred Eisenstadt and artist Rudolph Freund (Fig. 1).

**Fig. 2:**
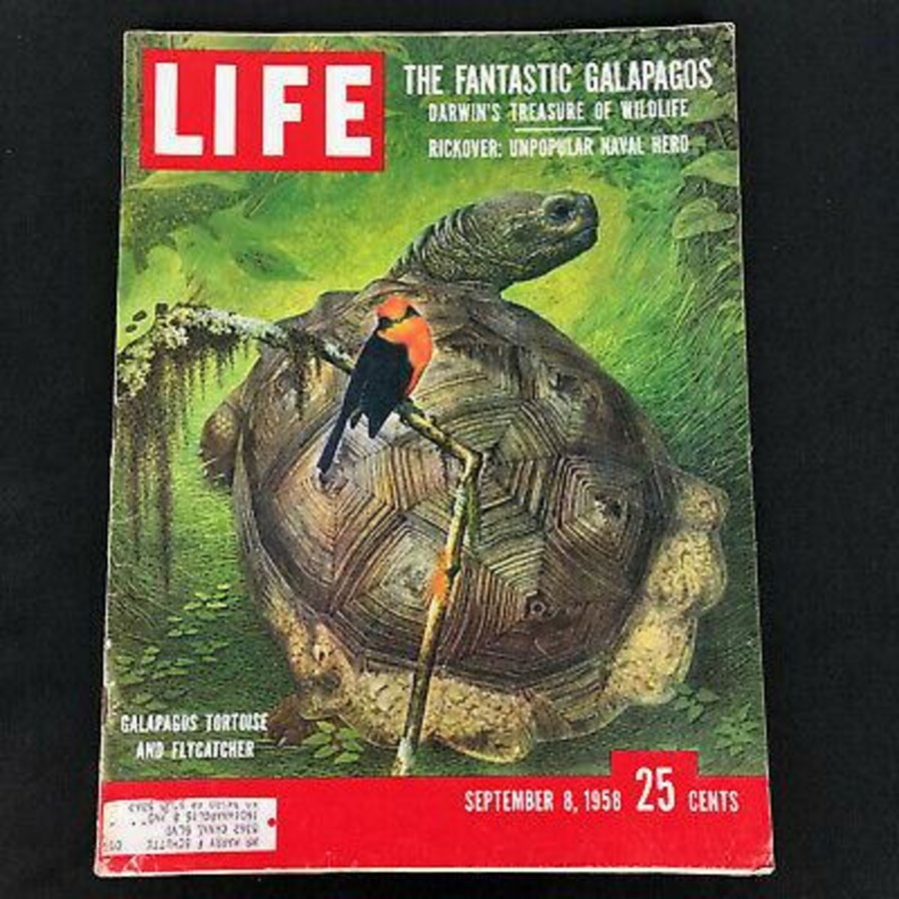
September 8, 1958, cover of *Life Magazine*. *Life Magazine*

Their four months in the islands resulted in two *Life* cover stories in 1958. One was written by Julian Huxley on June 30, 1958, on the “Life of Darwin,” and another on September 8, featuring a tortoise and flycatcher on the cover (Fig. 2).

Rudolph Freund and Alfred Eisenstadt created a photographic and artistic portrait of life in “The Enchanted Isles” and *Life* presented the two issues “to commemorate the 100th anniversary of a great turning point in human thought.”^12^ Such publicity, invoking enchantment and turning points in human thought, played a role in emerging interpretations of the Galápagos.

**Fig. 3:**
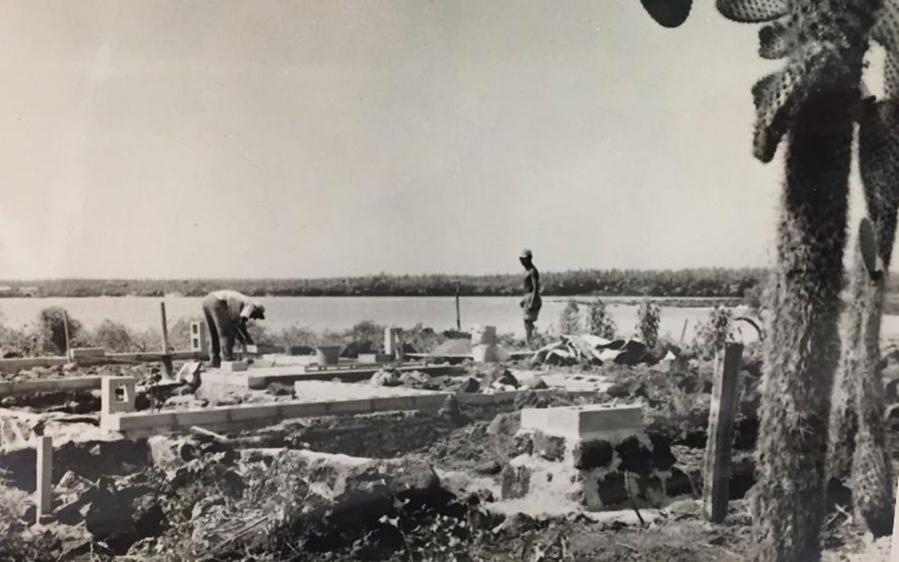
Building the station. “Organizational Materials,” Box 112, Papers of Julian Huxley, Rice University

On July 4, 1959, Ecuador issued an executive decree establishing an emergency law that declared the Galápagos to be “zones of reserve and National Parks” and officially recognized the planned Charles Darwin Research Station, to be administered by the Belgian fund-raising entity the Charles Darwin Foundation. Dedicated in 1964, the new station contained a laboratory for about ten scientists, with a dark room and washing and toilet facilities. In April 1964 the station’s new research vessel the *Beagle* arrived in the Galápagos, providing a seagoing vessel for travel from island to island (Fig. 3). The station had an annual income in 1964 of around $15,000.^13^

Just as earlier efforts to preserve and protect the islands were linked to the centennial of Darwin’s visit, this one was linked to the centennial of his publication of the *Origin of Species* in 1859. As Vassiliki Betty Smocovitis showed, the anniversary of 1959 was a chance to promote a unified science of evolution which established positivism as an organizing principle for all biological work. It was, she says, a “perfect opportunity to establish once and for all for wide audiences the facticity of evolution by means of natural selection” (Smocovitis 1999, p. 279). Huxley was a leading proponent of this idea and even a promoter of evolution as a replacement for religion. John C. Greene has noted that it was Huxley’s ambition from youth onward “to define a world view based on evolutionary biology that would unite mankind under the banner of evolutionary humanism and displace forever the creeds and dogmas that had ‘retarded the progress of civilization’ in past ages” (Greene 1990, p. 39; Phillips 2007). “Civilization,” understood in hierarchical terms, played many roles in the creation of the Galápagos park and station.

## Disciplining Scientists

From the very beginning, CDRS staff were worried about the behavior of visiting scientists. In late 1963, just as a major, celebratory CDRS expedition, the Galápagos International Scientific Project (GISP), was expected, then director of the research station David Snow expressed concerns about what the visiting scientists were likely to do during their visit.^14^ In a letter announcing his resignation (because his pregnant wife did not want to stay in the Galápagos) Snow mentioned that he would

like to ask one more thing, unrelated to the main subject of this letter. In addition to the populations of Saddleback tortoises in Santa Cruz, I have now found what appears to be a very small population of land iguanas living on Hood, an island where no land iguana has hitherto been found. It may well be that the participants in the G. I. S. P. understand that such animals are not to be collected. I wonder if the organizers of the project have this clearly in mind. It would be disastrous if participants arrived with the idea of collecting for their local museums; the collecting instinct is so strong in many American biologists.^15^

Snow’s fears that the privileged participants in the GISP might not understand that rare iguanas were “not to be collected” captures a professional reality: Many biologists—not just ones from the United States—were unaccustomed to being constrained in their collecting practices, particularly in remote field sites where rare species might be encountered.

When Eibl-Eibesfeldt returned for a visit in 1965, his reflections reinforced Snow’s concerns in 1963. He confirmed that the existence of the new station had increased scientific work in the islands as “more and more the Station attracts visiting scientists from all over the world” but this “very positive fact … creates some problems we should be aware of.” Seeds attached to the clothing of scientists “are transferred to other places. Small insects might go with them.” Such risks might be unavoidable “but more serious inter-island transfer could be prevented. Only recently a distinguished herpetologist on a scientific mission kept marine iguanas from Hood, land-iguanas from the South Plazas Island and Tropiduri from various islands on Santa Cruz (Indefatigable). Animals of all three species escaped through negligence.” (Eibl-Eibesfeldt 1965, pp. 25–26). Other reports of inter-island transfer of species as a result of scientific work had also been received. If the “peculiarity of the Galápagos is to be preserved” certain precautions are a necessity, he said, and “only with the special permission of the director of the station should any scientist be allowed to transfer animals from island to island” and, moreover, precautions to prevent the escape of such animals “must be taken.” He wrote: “[t]he wingfeathers of birds should be clipped; the entrance to cages should have two doors the first to be closed behind the caretaker before the second is opened, to mention some precautions.” Most importantly, he said “we certainly want to avoid the situation that scientists become a greater threat to the fauna than the native population” (Eibl-Eibesfeldt 1965, p. 26).

Reflecting these issues, a blank 1967 contract form for a visiting scientific expedition suggests in its restrictions some of the more difficult behaviors. The form said that conservation “must have absolute priority over all other considerations,” and that those directing expeditions must “conform at all times to these interests of the foundation.” The expedition leader would “abide by regulations protecting designated zones or species and by all obligations resulting there from, unless special permission to the contrary has been received.”^16^

This generic male scientific director would “not disturb protected habitats nor collect any animals or plants other than those for which he has obtained special collecting permits” and would recognize “the delicate balance which exists amongst the native species and the increasing rarity of many of them.” He would not collect specimens in large numbers, and would “avoid completely those whose skins would contribute nothing new to science.” He would also “avoid unnecessary disturbance to colonies of birds and reptiles and … refrain from any activity which might lead to modification in animal behavior.” Only with “special permission of the president” (of the Foundation) would this scientific leader “transport any plant or living animal from one island to another.”^17^

Furthermore, visiting “foreign” scientists should consult only with the officials of the Brussels-based Charles Darwin Foundation, and not with local people, journalists, or Ecuadorian scientists. The form states that “the head of the expedition shall conduct official correspondence concerning the expedition *only* with the president of the foundation, the secretary general, the director of the station, their duly authorized representative or his own immediate superior authority, and if writing to other than the director should furnish him with a copy of his correspondence.” Expedition leaders should not make any request to “any Ecuadorian authority” nor should they say anything to the press without the permission of the director of the research station.^18^

Questions about what kinds of research should be prioritized also arose quickly in the first few years of the station’s operation. In the 1969 station publication *Noticias de Galápagos* a report on scientific priorities stated that it was “not the Charles Darwin Foundation’s policy to attempt to exercise close control over investigation carried out in the Galápagos by visiting scientists,” but such research should be “compatible with the aims of” the foundation. “If it becomes necessary to allocate limited laboratory space among a number of different applicants with different research programmes, a choice must be made,” the report said, and preference would be given to those whose work was “directed at evolutionary problems in general or problems relating to the evolution of the Galápagos flora and fauna in particular, rather than to problems which could be equally well investigated elsewhere.”^19^

Some resented the new rules the CDRS imposed, and in 1974 Harvard primatologist Coolidge proposed that the CDRS adopt the well-known academic strategy of outsourcing blame to advisory committees. He advocated creating a “small advisory committee for Europe and another one for the new world” as a “safeguard, to deal with difficult situations. Bob Bowman has always been a thorny person in dealing with Galápagos matters. His important research on Darwin Finches has justified giving him a special VIP status, but this does not excuse his by-passing the procedures which we have established to control the export of Galápagos species that need to be protected against scientific exploitation.”^20^

**Fig. 4:**
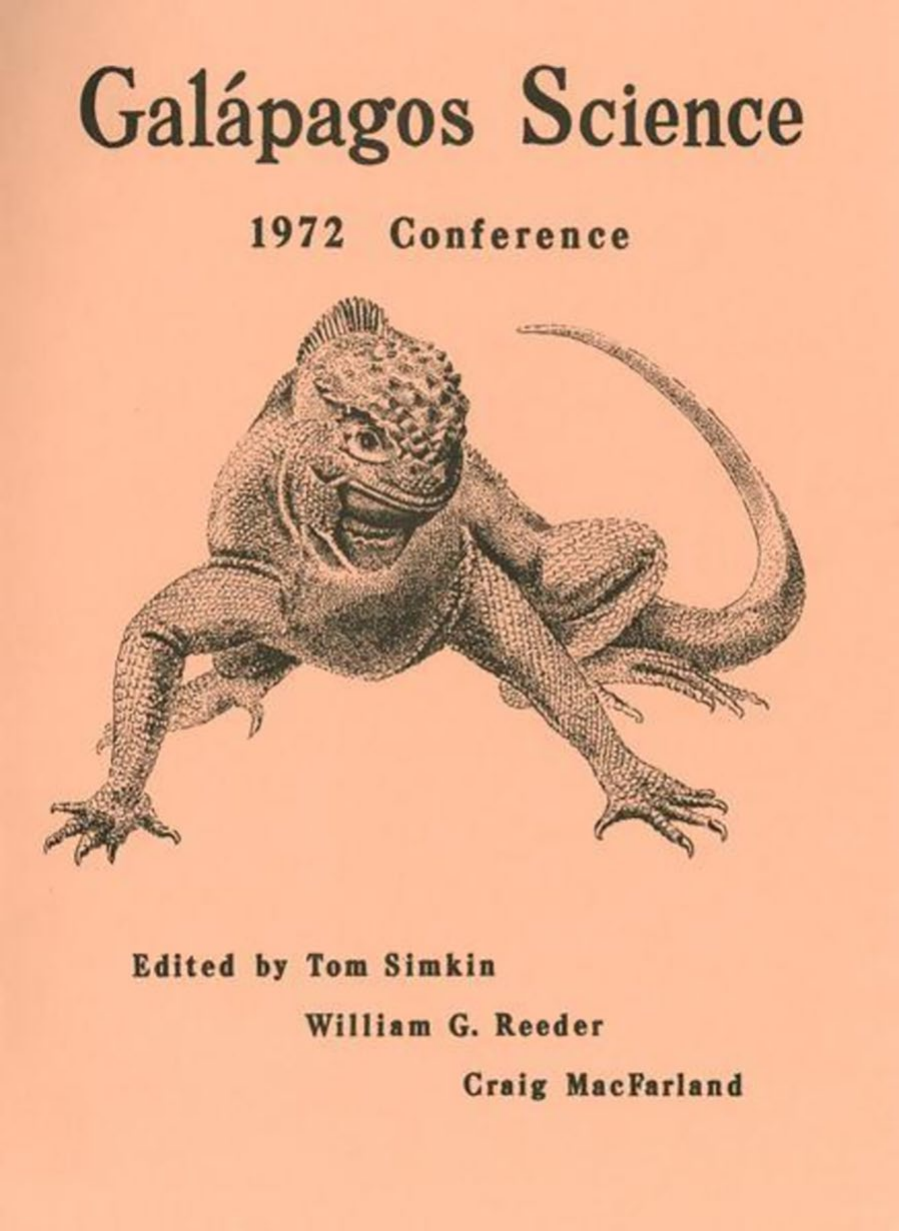
Cover Image, Final Report of the Galapagos Science Conference, Galapagos Science: 1972 Status and Needs: Report of Galapagos Science Conference, October 6–8, 1972, Washington D. C. From: University of Wisconsin Library, https://search.library.wisc.edu/digital/AC4LIVN67KQVVJ8X

The procedures he referred to had been outlined in the 1972 recommendations of the Galápagos Science Conference. This group, 36 scientists from different disciplines all of whom were familiar with the Galápagos, met at the Smithsonian Institution in Washington DC in October 1972, with a mandate to establish priorities for both conservation research and “fundamental scientific investigation.” The number of scientific missions visiting the islands had increased from six in 1965 to seventeen in 1971 (Simkin et al 1972, p. iv) and the increase was taking a toll on both staff and physical spaces. Participants at the conference told a tale of scientific urgency, outlining all the deficiencies of data collection in the Galápagos, describing significant gaps in plant ecology, systematics, oceanography, geology, paleoecology, ornithology, and general biology (Fig. 4). The islands had been studied haphazardly, their collective account suggested, and even the fishing resources in the region, of some importance economically, were not understood (Simkin et al 1972). All of this ignorance and lack of data was a little confusing to some participants, given the islands’ “justifiable status as holy land for evolutionists” (Simkin et al 1972, p. 67).

In a list of “Threats to the Galápagos Environment,” the report noted that it was “vitally important that scientists demonstrate sound conservation principles in the islands. Though the total damage has been minimal, some scientists have disturbed animal populations, allowed campfires to get beyond control, denuded some areas of wood for campfires, and left extensive amounts of litter” (Simkin et al 1972, p. 70). Such infractions could hurt not only the islands, the report stated, but the CDRS itself.

A lengthy Appendix III, prepared by Field Station Director Peter Kramer and Tom Simkin, Secretary for the Americas at the Smithsonian, provided “Information for Scientists Staying at the Charles Darwin Research Station.” This appendix explained the “list of requirements for scientists set by the National Park and Darwin Station” (Simkin et al. 1972, pp. 77–101):

Don’t bring any live material from the continent to the Archipelago or back and don’t carry any live material from island to island. Keep your influence on the environment to an absolute minimum: Don’t make campfires (Bring a kerosene stove; you can buy one in Guayaquil.) Don’t leave litter. (Simkin et al. 1972, p. 77)

Kramer and Simkin warned that some proposals could be turned down by CDRS, and one factor shaping the acceptance of projects was possible harm: “While most field research leaves some small temporary mark on the environment, the Park administration and the Darwin Foundation will not permit projects that will do serious harm to any element of the indigenous fauna or flora” and “collecting of specimens not related to the project in question will not be permitted” (Simkin et al. 1972, p. 78). Describing “responsible scientists” as those “aware of the extreme care needed to prevent the artificial intermingling of island populations,” they emphasized that even ticks, burrs, and soil in rubber soles could be “a great hazard to biotic integrity” (Simkin et al. 1972, p. 84).

Later the supposedly thorny Bob Bowman himself complained to Coolidge that he was “not at all certain that I like the direction that the Darwin Station is moving in, especially as it becomes a ‘home’ for visitors to squat for inordinately long periods of time. I always perceived that installation as a ‘field’ station out of which scientists operated, and not a base at which research analyses should be carried out, work that could more effectively be done at one’s home institutions. The number of staff members is almost staggering. Dr. Henrick Hoeck’s role as Director of the CDRS is an impossible job!”^21^

There were other “outsider” concerns about the station and the behavior of scientists. Apparently some local residents believed that the “scientists rather than the settlers in the islands did most of the killing,” hunting tortoises, birds, and other animals in ways that undermined the goals of the park. In a 1974 report, Corley-Smith, former editor of *Noticias*, and then Darwin Foundation Secretary-General, 1972–1982, noted that “[h]owever unjustified these allegations may be I think that we should always bear in mind that these suspicions exist especially when we are considering projects from visiting scientists. This is not just a philosophical question of whether scientists have a plenary indulgence to do things that they would denounce as crimes if committed by anyone else: this is the immediate problem of preserving the image of the [Charles Darwin] foundation in Ecuadorian eyes.”^22^

Others also suggested that the scientists affiliated with CDRS were damaging wildlife: In 1975, then Station Director Craig G MacFarland reminded Corley-Smith about a letter sent by Dr. Norman Hickin to the *Times of London*.^23^ In the published letter, Hickin complained about the banded birds and the painted turtles he encountered on a research trip to the islands. He was, he said, “amazed, horrified and disgusted to find that a high proportion of the endemic birds, the waved albatross, the flightless cormorant, the Galápagos penguin, the Galápagos hawk and other species carried metal straps around their legs. One mockingbird had the whole of its breast painted blue. A number of the iguanas were seen with pieces of colored plastic apparently sewn into their backs.” CDRS, he said, should “take steps to prevent the contamination, *by scientific personnel accredited to them,* [my emphasis] of the very wilderness they have undertaken to respect.”^24^

This letter annoyed CDF leadership, which responded publicly to criticize Hickin. But three years later that initial CDF response bothered MacFarland. He wrote: “You may remember that Dr. Norman Hickin wrote a rather peevish letter to the Times about the conduct of scientists in the Galápagos (Ringing, tagging, dyeing, etc.). Peter Scott and David Snow replied and Roger Perry and I handled Dr. Hickin pretty roughly in a radio discussion. This seemed necessary at the time but I have felt vaguely uncomfortable about it ever since as some of his strictures were justified and, as a matter of record, do seem to have been accepted in the [new] Master Plan.” As a result of these regrets, MacFarland said, “we are promoting his [Hickin’s] new book *‘Animal Life of the Galápagos*.*’* I do not know how Dr. Hickin, an entomologist, ranks in the scientific world, but in commerce he created our leading wood-worm extermination and timber treatment firms.” He asked Corley-Smith to share mailing lists for Ecuadorian sources and South American contacts for marketing the book.^25^ Hickin’s complaints, initially rejected, had become consistent with the new management plan—and promoting an unrelated book served as a public apology for earlier attacks.

In 1973 Corley-Smith suggested that the research station was also being badly used—or underused. He noted that then director Peter Kramer wanted to expand and add married quarters for visiting scientists, but during his recent one month visit the twentybed dormitory and the dining room were mostly empty. “It seems to me that too much building might create an impression of affluence which would be misleading; and there is already a striking contrast between the standards of the station and those of the village—which incidentally has expanded as rapidly as the station.”^26^

Another recurring problem involved the research vessel, the *Beagle*. Beginning in 1963, the CDRS maintained a series of scientific field boats, all named Beagle, and by this time *Beagle III* was in need of maintenance and repair—the radar was broken and Corley-Smith reported that no other boat in the islands had radar, and no one knew how to repair it. But the real problem was that it was not being used by scientists, because at $100 per day, plus mileage and food, the daily fee was too high. Scientists rarely used the ship, preferring to travel “more slowly and in less comfort but much more cheaply in a fishing boat.” The ship’s laboratory had become a storage space, and the ship was in port more than necessary. Hunters sent out to control introduced animals, supported by the World Wildlife Fund, were the primary users of the ship, so the World Wildlife Fund was “footing the bill” and subsidizing the *Beagle*.^27^

In its first decade of operation, the research station struggled with increasing costs (especially for the Beagle), new questions about how to manage and organize scientific work, and continuing tensions in relationships with residents and Ecuadorian officials. It also struggled to negotiate with sometimes-resentful scientific visitors, who were competing for space and attention and required to follow strict rules in the field.

## Conclusions

The Charles Darwin Research Station was located in a place understood to be a site of “global heritage” and scientific prestige—a site of almost religious significance. The Galápagos were among the first UNESCO World Heritage Sites identified in 1978. Indeed, they were probably the first, because in the numbering system they are identified as “1.” And their recognition barely left a paper trail: As Lynn Meskell shows in her study of UNESCO and World Heritage, they were approved with almost no discussion, no complex procedures, no paperwork, “enshrined with scarcely any justification or documentation” alongside Yellowstone National Park and the pyramids at Giza (Meskell 2018, pp. 71–72).

**Fig. 5:**
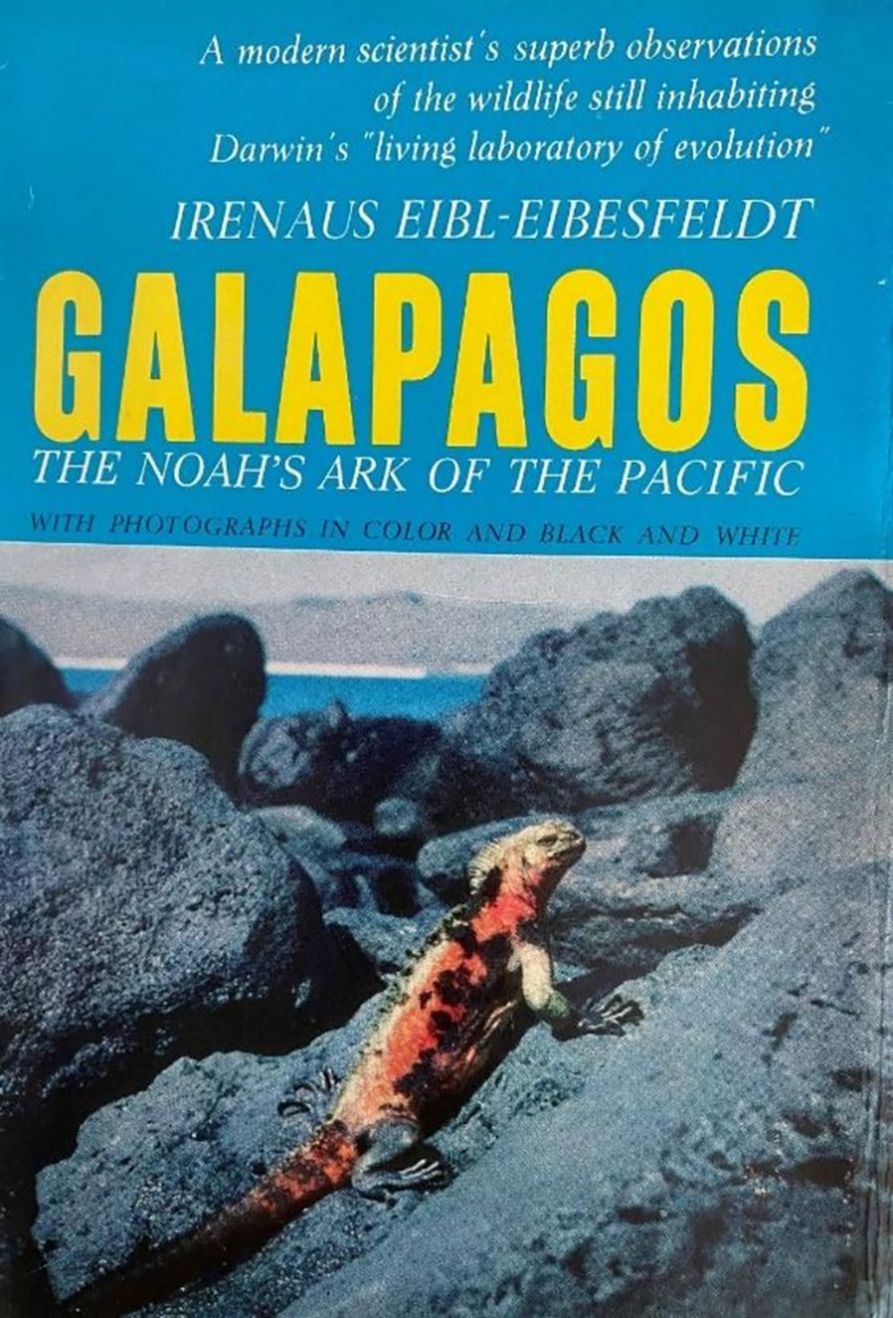
Cover of Eible-Eibesfeldt’s 1958 book. Photo of book cover by author

They were at one point on the list of possible sites for US nuclear testing grounds, though military planners ultimately settled on the Marshall Islands (Weisgall 1994, p. 32). Instead of being destroyed, the Galápagos were preserved as sites for secular pilgrimage, with Julian Huxley’s evolutionary humanism invoking their unquestionable beauty as evidence of the spiritual qualities and deep meanings of evolutionary theory.^28^ They were also commonly described in the 1950s and 1960s as “Noah’s Ark” (Eibl-Eibesfeldt 1958, 1961; Vonnegut 1985). For Huxley and many others, the islands were sites equivalent to the hallowed pilgrimage sites of Christianity, holy places of secular worship, which were at risk of being destroyed by the presence of life (human and non-human) that did not belong there (Fig. 5).

Outward-facing texts produced by the station and its fund-raising entity, the Charles Darwin Foundation, commonly sought support by describing problems posed by the growing number of residents in the islands, the fishing industry, and the goats, rats, cats, and other invasive species that threatened local flora and fauna. These were real problems, but they were not the station’s primary responsibility. In public appeals for money, I have not found any discussions of the difficulties of controlling European or American scientists, or any self-congratulation from station officials that they had kept unscrupulous or unreliable scientific researchers out of the islands. In a letter sent to possible donors in 1968, for example, the problems were summarized with a focus on those (apparently lawless) Ecuadorians living in the Galápagos:

We and the government of Ecuador are faced in the Galápagos with an expanding population of humans existing marginally who find it necessary for their basic existence to make catastrophic inroads on an ancient biota of small magnitude and extreme tenderness, and almost completely isolated from law-enforcement agencies. Your small contribution will help us educate Galapaguenos but only your voices combined with those of our European, Latin American, Asian and other friends can solidify a world of public opinion that cannot go unnoticed.^29^

This was signed by J. Laurens Barnard, Secretary for the Americas for the Charles Darwin Foundation.^30^ The primary enemy to be controlled and educated in this text was the local human population—the Galapaguenos.

This claim echoed perspectives at other reserves and national parks around the world. Ashanti Shih’s analysis, for example, of the creation of a national park in another volcanic island group, Hawai'i, explores the park as a “perfect laboratory for U.S. experts to study ‘curious’ flora, fauna, and geological processes” and a space that excluded non-scientists. This way of seeing parks “has ramifications for how we think about issues of access and justice,” as “the parks not only barred certain peoples and their ways of life, but also provided access to scientists” (Shih 2019, p. 5). Julia Weiskopf’s study of Huxley’s “wildlife diplomacy” points out that “working with wild animals on the international stage was a complex strategy, as officials confronted the thinly-veiled racism of conservationists who doubted the commitment and capacity of African-run states to serve the natural world” (Weiskopf 2015, p. 433). Hennessy in her study of the Galápagos observes that “plans for a new global empire based on rational scientific management were shot through with paternalistic racial and cultural elitism. UNESCO and the IUCN were among the postwar institutions that comprise the new imperialism, premised on internationalism and faith in the expansion of capital, science and technology to spread modern Western values” (Hennessy 2019, p. 121; see also Hennessy 2018). Similarly, Lino Camprubi considers the creation of *another* Biological Research Station, in the same year as the Galápagos Station, and mediated through some of the same networks orbiting Julian Huxley. In fascist Spain (rather than in the recently decolonized world), it was a natural space appreciated particularly by hunters—popular as a pristine hunting grounds for wealthy European trophy hunters, and called by one “more African than Africa” (Camprubi 2020, p. 438). For me, the intriguing part of this case involves the hunters. Did research station authorities, who decided where and when scientists could engage in fieldwork, also select who could hunt and where? Camprubi’s focus is elsewhere, but the case is provocative in terms of understanding the real day-to-day work of these kinds of facilities (Camprubi 2020). Who exactly was being controlled?

Kramer’s own perspective in 1972 was that the station’s duties centered on keeping foreign investigators in line: “I look upon the Darwin Station primarily as an institution serving the development of science and the promotion of scientifically based environmental policies in Ecuador and all of Latin America. Scientists from developed countries play a decisive part in the realization of these objectives, but there is the danger that the Darwin Station devotes too much time and effort toward research projects that may be of interest to foreign scientists and institutions but are irrelevant to current problems of the National Park.”^31^

The regulatory control of field research is today fully institutionalized. Any expert in current-day planning to collect materials from almost anywhere in the world faces a system of permitting, registration, limits, and rules determining what they can take, what standards they must satisfy, how they can transport it, and what they have to do with it. Violations of these rules can come with criminal penalties and international prosecution. The historical pattern of free-wheeling autonomy in the biological field is today long gone.^32^ But in 1964, despite some existing rules and regulations, it was decidedly not, and the Galápagos Islands became a special test case for negotiating changes in long-standing field practices.

As Hennessy points out, the unfettered collection of rare and endangered species has been “easily edited out of the way that conservationists remember the past,” noting that early 20th-century collectors could simultaneously warn of the imminent extinction of tortoises, and then claim they had collected the last specimen (Hennessy 2019, pp. 69–70). An element of this easy editing played a role in the Galápagos Field Station’s public presentation.

For those promoting the station, calling attention to the damage done by local residents and fishing crews, and eventually tourists, was acceptable and indeed common practice. Calling public attention to the potential or actual misdeeds of scientists might undermine the legitimacy of the station itself, which depended on the support of both local residents and the scientific community. Yet I would suggest that acknowledging such issues could have played a role in generating local public support and understanding. Residents seem to have experienced scientific visitors as threats and enemies, and more straightforward and open communication about how scientists were (like everyone) expected to adopt strict conservation values in the islands might have ultimately facilitated the work of the research station.

Conservation programs have been critiqued for their relative silence around the priorities and needs of local and Indigenous groups. These silences were in my case joined by a matching silence around the awkward work of restraining visiting scientists. Essentially I have tracked some practices that “civilized” scientific elites, and propose that a careful consideration of the public and private narratives of conservation—of what is openly discussed and what is kept behind the curtain—raises important questions about the history of both fieldwork and conservation.

## Footnotes

1 Frank Sulloway’s work demonstrated that Darwin did not develop his theory of natural selection as a result of his brief, confused visit to the Galápagos Islands in September and October of 1835 nor did his ideas depend on the famous finches. Contrary to the legend, “Darwin’s finches do not appear to have inspired his earliest theoretical views on evolution, even after he finally became an evolutionist in 1837; rather it was his evolutionary views that allowed him, retrospectively, to understand the complex case of the finches.” And the finches, of course, are not even mentioned in Darwin’s 1859 *On the Origin of Species* (Sulloway 1982a, p. 32; see also Sulloway 1982b, 2009).

2 For a clear picture of how the Charles Darwin Foundation continues to explain its work, see its website: https://www.darwinfoundation.org/en/ (Accessed June 27, 2024).

3 The differing roles of “foreigners” and locals in this system reflect what I have characterized in a different context as a “citizenship credential”.

4 Most early official visitors to the islands were more interested in mapping and geology than in collecting biological specimens. The islands had never attracted a traditional Indigenous population. While Polynesian explorers in the Pacific—the *Vikings of the Sunrise* in the words of Maori scholar Te Rangi Hīroa (1938)—followed migrating birds to settle every island group in the vast Pacific, including distant, tiny Easter Island, the bird-rich Galápagos islands were overlooked or left behind (Te Rangi Hīroa 1938, 2024). A written record of discovery was generated by Europeans in 1535, when a Spanish bishop from Panama was blown off course during a royal mission to Peru and wrote home describing the islands as “hell” (Kramer 1983; Slevin 1959). Slevin’s meticulous 1959 history of the islands, which incorporates many primary sources (including the diaries of buccaneers and midshipmen), can be read in digital form at https://whalesite.org/galapagos/1959_Slevin_Galapagos_Exploration.htm.

5 These included the Lacey Act of 1900, the North Pacific Fur Seal Convention of 1911, the Migratory Bird Treaty Act of 1918, and the Bald Eagle Protection Act of 1940 (Benson 2012, p. 39).

6 “Scientist would set aside islands as wildlife sanctuary: Urges use of Galápagos.” March 21, 1933, *The New York Times*, 15.

7 “Who were the CDF founders and why did they succeed, and others failed?” Charles Darwin Foundation. Last modified March 21, 2023. Accessed July 4, 2024. https://www.darwinfoundation.org/en/news/all-news-stories/who-were-the-cdf-founders-and-why-did-they-succeed-and-others-failed/.

8 A timeline and summary of the World Wildlife Fund is posted on the WWF site, https://www.worldwildlife.org/about/history; see also Camprubi (2020, pp. 437–441), Robinson (2005), Meskell (2013) and Xinsheng et al. (2015).

9 The scholarly literature on mid-twentieth century conservation and natural history is vast, and my key actors in this paper, particularly Harold Coolidge, Victor van Straelen, and Julian Huxley, appear in much of it. Some key studies include Barrow (1998, 2000, 2009); de Bont (2021); Dunlap (1988); and Davis (2007). See Huxley's own account, 1961.

10 Nicknamed Renki, and later an important innovator in the use of film to understand the biological underpinnings of human behavior. See Schiefenhövel (2018). https://ishe.org/2018-2/heb-vol-333-special-issue-to-commemorate-the-life-of-irenaus-eibl-eibesfeldt/obituary/.

11 An account of his experiences on this trip is in Eibl-Eibesfeldt (1961). See also Eibl-Eibesfeldt (1958).

12 “The fantastic Galápagos: Darwin’s treasure of wildlife.” 1958. *Life Magazine*, June 30 and September 8.

13 The first director of the planned station, the 28 year-old Swiss ornithology student Raymond Leveque, arrived knowing very little about the realities on the ground. He was quickly overwhelmed. There was no wharf to unload supplies, no roads across the arid stretch of cactus and scrub to the building site, no water supply, and no electricity. See Corley-Smith (1990, p. 10); and see Hennessy (2019, pp. 133–142). Charles Darwin Foundation for the Galápagos Islands: A progress report. 1963. See also Box 112, Folder 1, Papers of Julian Huxley, Rice University. For an account of Ecuadorian priorities in this process see Sevilla, 2017.

14 Snow’s stint as Director at CDRS was relatively brief, from 1964 to 1965, but he served on the Charles Darwin Foundation board after his return, and with his wife Barbara, also an ornithologist, carried out important studies of birds in the UK. David Snow later served as president of the British Ornithologists’ Union. See “David Snow,” *The Telegraph*, February 17, 2009. https://www.telegraph.co.uk/news/obituaries/4682464/David-Snow.html.

15 David Snow to Victor Von Straelen, August 17, 1963, Box 35 General Correspondence June to December 1963, n.d. 1963. Papers of Julian Huxley, Rice University Archives.

16 This blank form is in “1968 Institutions Association” in Box 42, of 45 HUG FP 78 14. Papers of Harold Coolidge, Harvard University Special Collections.

17 This blank form is in “1968 Institutions Association” in Box 42, of 45 HUG FP 78 14. Papers of Harold Coolidge, Harvard University Special Collections.

18 This blank form is in “1968 Institutions Association” in Box 42, of 45 HUG FP 78 14. Papers of Harold Coolidge, Harvard University Special Collections.

19 “Scientific Programme of the Charles Darwin Foundation” Noticias de Galapagos 14, 1969, pp. 13–21, 13. See also report by David Snow, Box 42 of 45 HUG FP 78 14 File 1968 Institutions Assoc 1968 report by the Charles Darwin Foundation. Some quotations from this report in same box and file: “Charles Darwin Foundation for the Galápagos Isles: Scientific Programme.”

20 Hal Coolidge to G. T. Corley-Smith, May 20, 1974, Box 18, File, HUGFP 78 16 File labeled 1974, Papers of Harold Coolidge, Harvard University Special Collections.

21 Bob Bowman to Hal Coolidge. March 5, 1979. Box 18, File D, Darwin, Charles Foundation for the Galápagos Islands, 1974, HJC Trustee, Papers of Harold Coolidge, Harvard University Special Collections.

22 G. T. Corley-Smith to Jean Dorst, February 7, 1974. File D, Darwin, Charles Foundation for the Galápagos Islands, 1974, HJC Trustee, Box 18, Papers of Harold Coolidge, Harvard University Special Collections.

23 MacFarland was the Director of the Research Station 1974–1978, and President of the board of the Charles Darwin Foundation 1985–1996.

24 Hickin, Norman. “Galápagos Experiments,” *Times of London,* December 29, 1972, p. 11, from The Times Digital Archives, gale.com Document Number GALE|CS185300893.

25 G.T. Corley Smith to Craig McFarland (then director of the CDRS), April 14, 1975, Box 18, File D, Darwin, Charles Foundation for the Galápagos Islands, 1974, HJC Trustee, Papers of Harold Coolidge, Harvard University Special Collections.

26 G. T. Corley-Smith, by this time Secretary General for the CDF, reported on his visit to the field station in April and May of 1973, quotation is on page 2 of this report. Box 18 HUGFP 7816, 1973. Papers of Harold Coolidge, See also G. T. Corley-Smith to Jean Dorst, February 7, 1974. File D, Darwin, Charles Foundation for the Galápagos Islands, 1974, HJC Trustee, Box 18, Papers of Harold Coolidge, Harvard University Special Collections.

27 G. T. Corley-Smith to Jean Dorst, February 7, 1974. File D, Darwin, Charles Foundation for the Galápagos Islands, 1974, HJC Trustee, Box 18, Papers of Harold Coolidge, Harvard University Special Collections.

28 The implications and consequences of Julian Huxley’s ideas about evolution and humanism have been explored by many scholars, and yet there is still much, much more to do. See Greene (1990; Smocovitis 1996, 2016; Phillips 2007).

29 Undated, “Dear Friend” letter, in 1968 file. Box 42, Papers of Harold Coolidge, Harvard University Special Collections.

30 Undated, “Dear Friend” letter, in 1968 file. Box 42, Papers of Harold Coolidge, Harvard University Special Collections. .

31 Peter Kramer, Director’s Report, 1972. Library, CDRS, Santa Cruz, Galápagos.

32 Some relevant instructional websites include US National Park Service: https://www.nps.gov/yell/learn/nature/npsconditions.htm. Convention on International Trade in Endangered Species of Wild Fauna and Flora, CITES, enacted 1973: https://cites.org/eng/disc/how.php. US Fish and Wildlife Service: https://fwsepermits.servicenowservices.com/fws. There are many others.
